# The Role of the European Reference Network for Rare Bone Diseases (ERN BOND) and European Registries for Rare Bone and Mineral Conditions (EuRR-Bone) in the Governance of the Management of Rare Bone and Mineral Diseases

**DOI:** 10.1007/s00223-024-01256-7

**Published:** 2024-07-26

**Authors:** Ana Luisa Priego Zurita, Manila Boarini, Lorena Casareto, Mariya Cherenko, Marina Mordenti, Alice Moroni, S. Faisal Ahmed, Natasha M. Appelman-Dijkstra, Luca Sangiorgi

**Affiliations:** 1 Department of Internal Medicine, Division of Endocrinology, Leiden University Medical Centre, Albinusdreef 2, Postbox 9600, 2300 RC Leiden, The Netherlands; 2 Department of Rare Skeletal Disorders, IRCCS Istituto Ortopedico Rizzoli, Bologna, Italy; 3 Developmental Endocrinology Research Group, School of Medicine Dentistry and Nursing, University of Glasgow, Glasgow, UK

**Keywords:** European reference networks, Databases, ERN BOND, Rare conditions, Rare diseases, Registries, Governance

## Abstract

Rare diseases (RDs) bear a significant challenge to individuals, healthcare systems, and societies. The European reference network on Rare BONe diseases (ERN BOND) is committed to improving multidisciplinary, patient-centred care for individuals with rare bone and mineral diseases (RBMDs). Its affiliated project, the European registries for rare bone and mineral conditions (EuRR-Bone) collects data using two different platforms, an electronic surveillance system (e-REC) that captures the occurrence of RBMDs and the Core Registry, a platform with the infrastructure for collecting Core data fields and longitudinal generic and condition-specific information. With emerging registries and the overlap with other ERNs, it is key to maintain the capability of the platforms to adapt to the needs of the network and the community whilst adhering to quality and FAIR (findable, accessible, interoperable, and reusable) principles. This binomial ensures long-term sustainability and potential advances in the care pathway of RBMDs whilst promoting good practice standards within Europe and beyond.

## Introduction

Rare diseases (RDs) present a significant challenge to individuals, healthcare systems, and societies. Their complexity and low prevalence often result in delayed diagnosis, limited treatment options, and considerable economic strain. Strategic governance of RDs is essential for improving patient outcomes and reducing the overall burden [1]**.** The European Reference Networks (ERNs) were initiated in 2017 by the European Commission (EC), involving currently more than 1,600 specialised healthcare providers. ERNs aim to facilitate access to high-quality, cost-effective care for rare or low-prevalence complex diseases through collaborative efforts across Europe. With each ERN focusing on a specific medical domain of RDs or complex conditions, these virtual networks, totalling 24, enable knowledge sharing, care coordination, and the reduction of negative outcomes for patients. The ERN BOND—European reference network on rare BONe diseases—is committed to improving multidisciplinary, patient-centred care for individuals with rare bone and mineral diseases (RBMDs) (https://ernbond.eu/). By promoting rapid information exchange and involving patient representatives, ERN BOND strives to expedite diagnosis and treatment processes, thereby enhancing patient outcomes [2].

Concurrently, RD registries play a crucial role in improving patient care by facilitating data pooling and sharing, public health surveillance, and communication amongst multidisciplinary teams, including patients [3]. They also support ERNs activities, reinforcing epidemiological surveillance [4]. Whilst over 750 RD registries exist within Europe, the diversity in nature and governance criteria necessitates the development of guidance to maintain high-quality registries. The international registration of patients with RDs is encouraged by the European Union (EU), with initiatives like Orphanet (https://www.orpha.net/) and RD-Connect [5] aiming to identify existing registries. Despite the need for new registries, increasing awareness and participation in existing ones is pressing. The European Registries for Rare Bone and Mineral Conditions (EuRR-Bone), funded by the EU health programme, was established in 2020 to promote the collection of high-quality detailed information and to map the activity of centres caring for these conditions.

This article will explore essential aspects of data access and governance policies for RD registries, using EuRR-Bone as a model for promoting good practice standards conducive to long-term sustainability. Additionally, we will focus on the role of ERN BOND in governing the care of rare bone and mineral diseases (RBMDs).

### The European Reference Network on Rare BONe Diseases—ERN BOND

The ongoing mission of ERN BOND is to implement measures that facilitate multidisciplinary, holistic, continuous, patient-centred, and participative care provision to individuals living with RBDs, supporting them in the full realisation of their fundamental human rights.

**Fig. 1:**
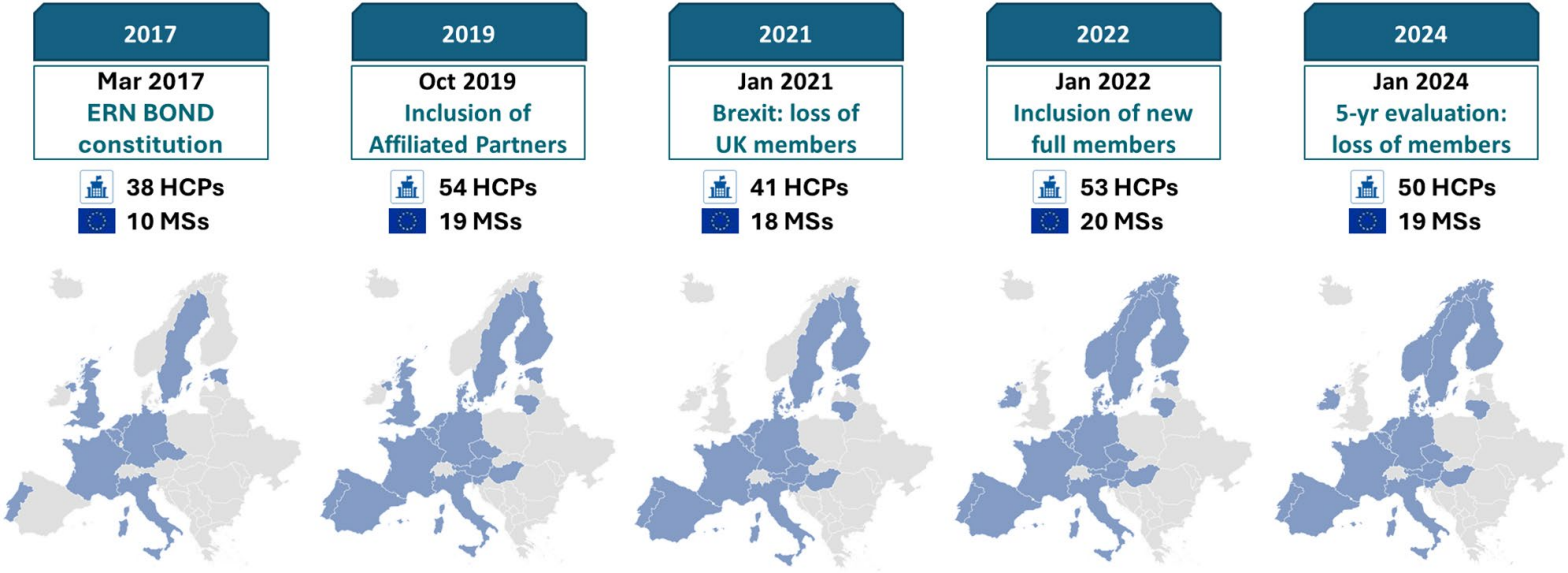
Change in the number and geographical distribution of ERN BOND members over time. HCPs: healthcare providers; MSs: member states

In 2017, ERN BOND comprised a network of 38 founding healthcare providers (HCPs), representing centres of expertise within the network’s scope, located across 10 member states (MSs). Since then, significant changes have occurred in terms of both network size and geographical coverage (Fig. 1). Currently, ERN BOND encompasses a total of 50 HCPs from 19 European MSs, comprising 44 full member HCPs and six affiliated partners (including two associated national centres and four national coordination Hubs).

**Fig. 2:**
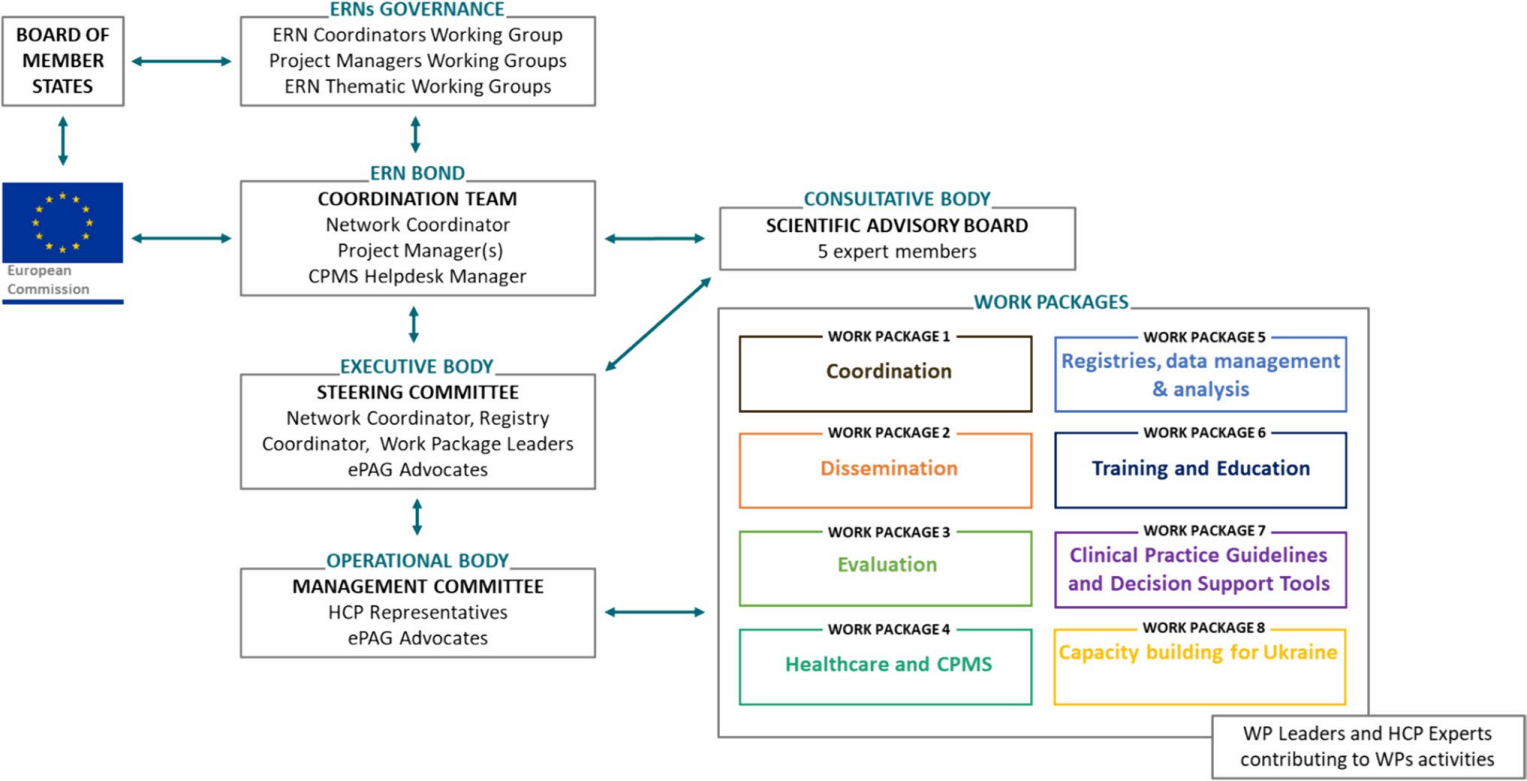
Governance of ERN BOND

ERN BOND established its governing structure upon its inception (Fig. 2). Unlike some other ERNs, the governance structure of ERN BOND is activity oriented rather than disease oriented, reflecting the unique nature of RBMDs. RBMD patients exhibit common special needs, and the disorders display significant clinical overlap and numerous sub-groups, necessitating frequent updates to clinical classifications (since 1970, 11 revisions of classifications have been issued) which complicates their organisation into sub-categories [6]. ERN BOND’s activities comprehensively address RBMDs and have evolved over time through thematic work packages (WPs). Each WP is led by an appointed leader responsible for managing and coordinating the respective scientific activities, with the involvement of clinical and scientific experts from ERN BOND HCPs, as well as representatives from European patient advocacy groups (ePAGs) working within the ERNs. These groups collaborate synergistically to advance the network’s objectives.

A devoted WP focusing on disease registry has been present since 2017. The outcomes produced by this WP contributed to building the original EuRR-Bone proposal [7, 8]. The network coordination includes the coordinator, who supervises the overall progress of all network activities and also acts as an intermediary between the ERNs governance bodies, the EC and other ERNs, and the coordination team, which carries out the day-to-day operational activities, as well as supports network members.

All WP leaders and two ePAG representatives are part of the Steering Committee, the decision-making body, chaired by the coordinator. The presence of ePAG representatives in the ERN BOND activities and in the governing bodies ensures that the patient’s perspective is always heard within the network. The Management Committee, the operational body, comprises a representative per HCP full member and all ePAG representatives.

Finally, ERN BOND can also rely on the scientific advisory board, which is the consultative body, providing scientific advice to the network regarding ongoing and upcoming priorities. Board members are experts in different areas, covering clinical research, diagnostic, ethical, legal, and social implications, ontologies, and patients’ representatives. They are nominated through voting by the management and steering committee members.

### The European Registries for Rare Bone and Mineral Conditions—EuRR-Bone

**Fig. 3:**
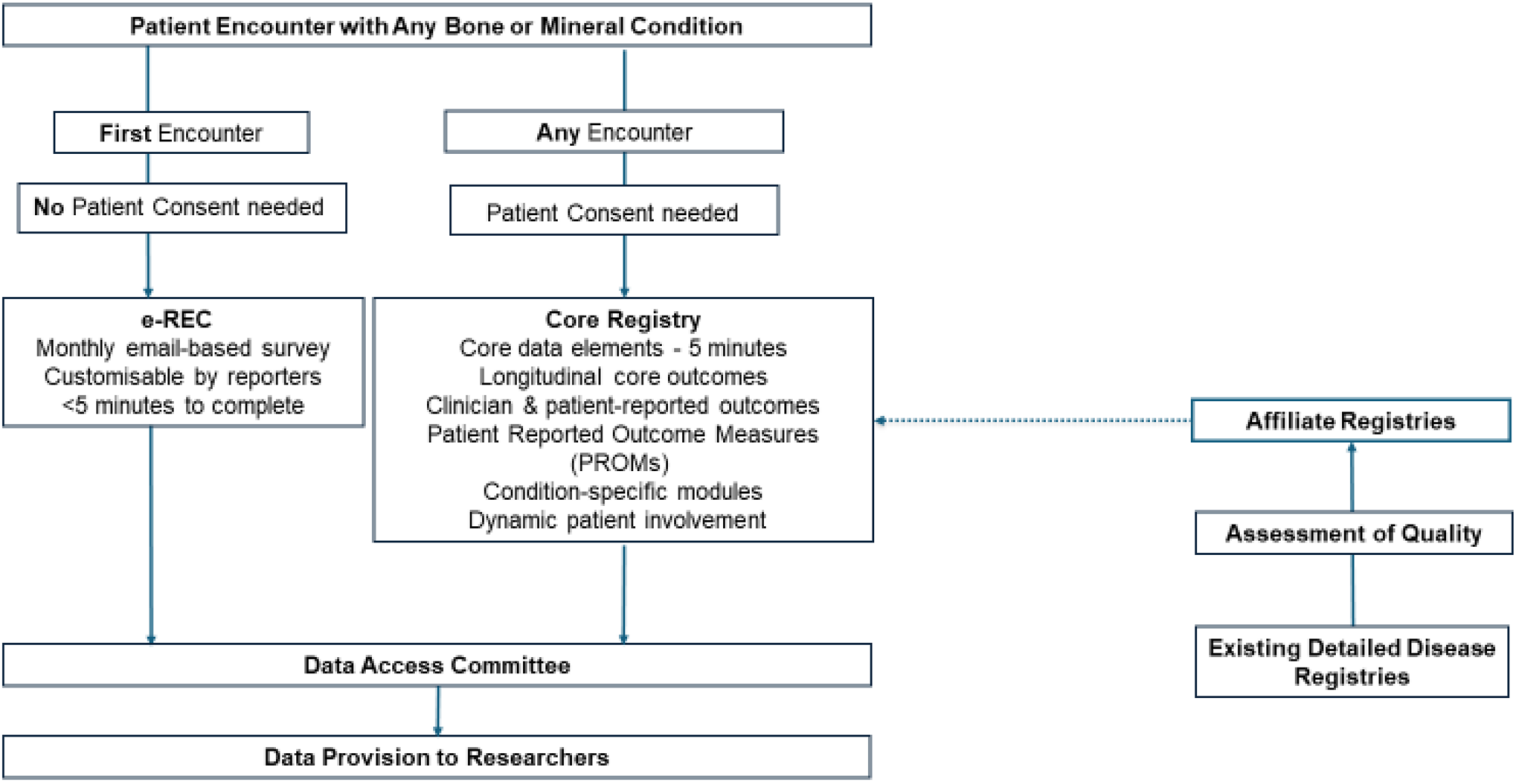
The EuRR-Bone project and its data collection platforms

In 2018, the first registries designed to support the needs of the ERNs were developed under a separate grant. Since 2023, these registries are incorporated within the ERN structure. It was foreseen that the registries would support the different needs of the ERNs by collecting data on the natural course of diseases. In 2020, the European Registries for Rare Bone and Mineral Conditions were established. Till then, only a few clinical and patient registries for RBMDs existed. These registries often lacked the long-term follow-up needed to characterise the natural history of these conditions, and the appropriate data structure to facilitate interoperability across registries. A survey disseminated by ERN BOND and addressed to healthcare professionals in Europe working with RBMDs showed that at that time there were no independent pan-European registries covering bone and mineral conditions [8]. Approximately, 42% of respondents reported other local or national initiatives, but these were of unclear maturity, size, follow-up, data quality and data depth, and with varying disease and geographical coverage. EuRR-Bone is the first pan-European registry for RBMDs. From the beginning onwards, EuRR-Bone had a close relationship with the European Registries for Rare Endocrine Conditions (EuRRECa), the registries designed to support the needs of the European Reference Network on Rare Endocrine Conditions (Endo-ERN). EuRRECa was established in 2018 and allowed EuRR-Bone in 2020 to embark on their existing platforms e-REC and the Core Registry [9] (Fig. 3)*.* These platforms are now, not only in use by members of both ERNs but also by non-ERN HCPs who have a special interest in RBMDs.

### e-REC

**Fig. 4:**
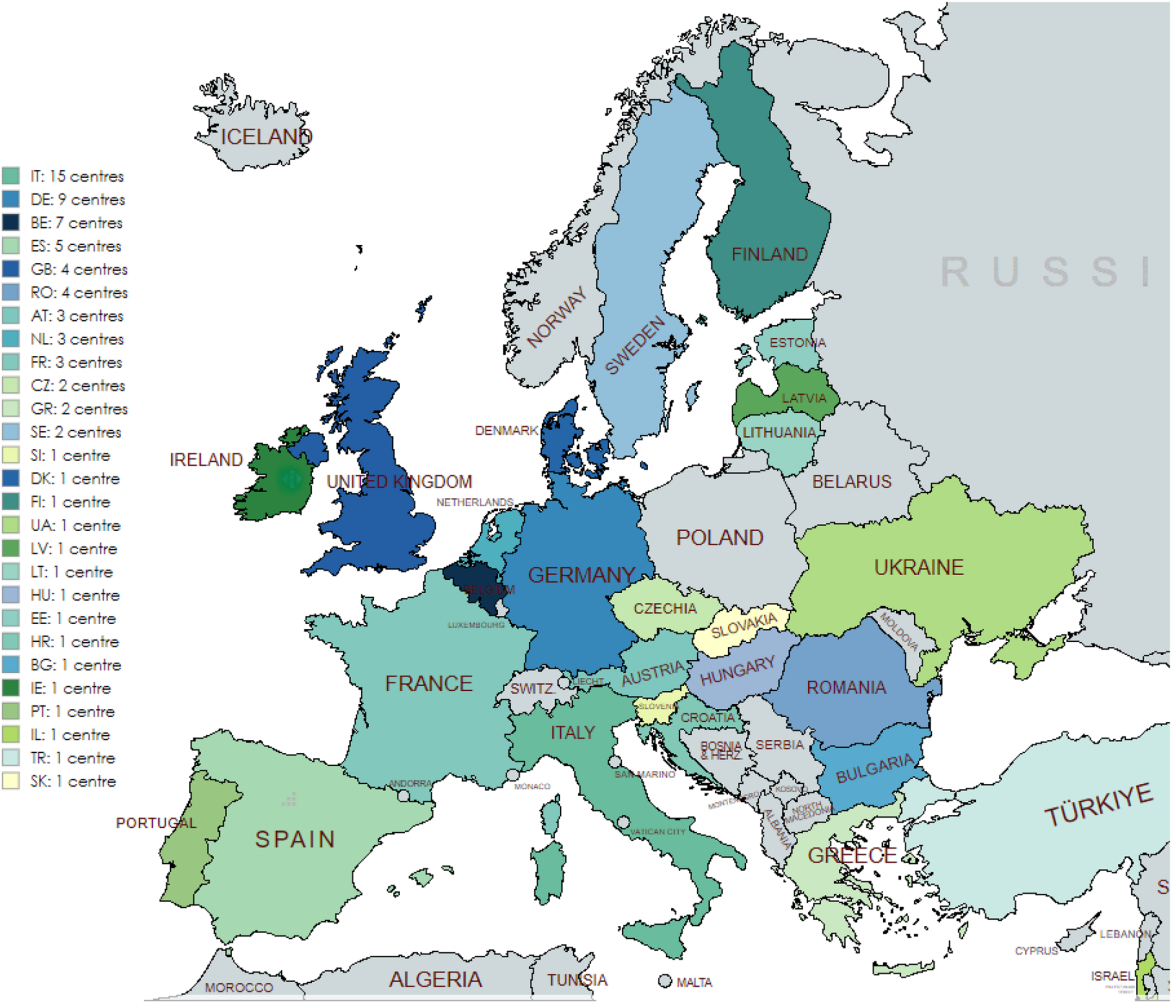
e-REC centres including ERN BOND and non-ERN BOND members

AT, Austria; BE, Belgium; BG, Bulgaria; CR, Croatia; CZ, Czech Republic; DE, Germany; DK, Denmark; EE, Estonia; ES, Spain; FI, Finland; FR, France; GB, Great Britain; GR, Greece; HU, Hungary; IE, Ireland; IL, Israel; IT, Italy; LT, Lithuania; LV, Latvia; NL, Netherlands; PT, Portugal; RO, Romania; SE, Sweden; SI, Slovenia; TK, Turkey; UA, Ukraine. Image created using MapChart.net.

The electronic reporting system, e-REC (https://eurreb.eu/registries/e-rec), allows continuous reporting of core activity indicators. This platform serves ERN BOND for its purpose of mapping the occurrence of rare conditions and members’ activity. Participating centres are invited to report on a monthly exercise the occurrence of any of the conditions covered by the network. Only the number of new cases is reported; thus, no personal identifiers are collected and no informed consent from the patient is required. e-REC is a simple yet flexible platform capable of adapting to emerging needs. For instance, during the COVID-19 pandemic, a project in collaboration with the European Society of Endocrinology (ESE) Rare Disease Committee, used the platform for a surveillance exercise where reporters were invited to notify the occurrence of a COVID-19 infection in patients with an existing bone or endocrine condition [10]. Until July 2024, 74 centres from 27 countries (three non-EU) had reported on the e-REC 4,071 new cases (2,900 in adults, 1,171 in children). Of these 74 centres, three are affiliated to ERN BOND, 24 to both ERN BOND and Endo-ERN, 33 Endo-ERN only, and 14 are unrelated to ERNs (Fig. 4).

### Core Registry

Registration of patients on this platform (https://eurreb.eu/registries/core-registry/) begins with obtaining informed consent from the patient. The requirements and process for obtaining local ethics approval to participate in this registry and data sharing policies may vary from centre to centre and across countries. The Core Registry collects a set of Core data fields, including the European standard common data element set for rare disease registration [11], allowing potential interoperability with other registries and databases, pivotal in rare disease research [12]. In addition to the Core data fields that are collected for every patient regardless of the diagnosis, condition-specific and patient-reported outcomes have been included within the Core Registry. Clinicians can access the platform, enter data, complete outcomes, and view not only their patients but also those entered by contributors from the same centre. Patients interested in accessing the platform will receive a link to create an account to access the Core Registry. Patients can view the data entered by the clinician and complete condition-specific and generic outcomes assigned to their role. Furthermore, they can change their language settings and data sharing preferences, including requesting the deletion of their data.

Condition-specific datasets have been developed by study groups composed of experts in the condition of interest. The groups include healthcare professionals from different disciplines and patient representatives. To date, seven condition-specific modules covering bone and mineral conditions have been installed in the Core Registry (https://eurreb.eu/registries/core-registry/condition-specific-modules/). This feature of the Core Registry provides an ideal platform for the characterisation of rare diseases, since it allows longitudinal collection of data via multiple data entries.

**Fig. 5:**
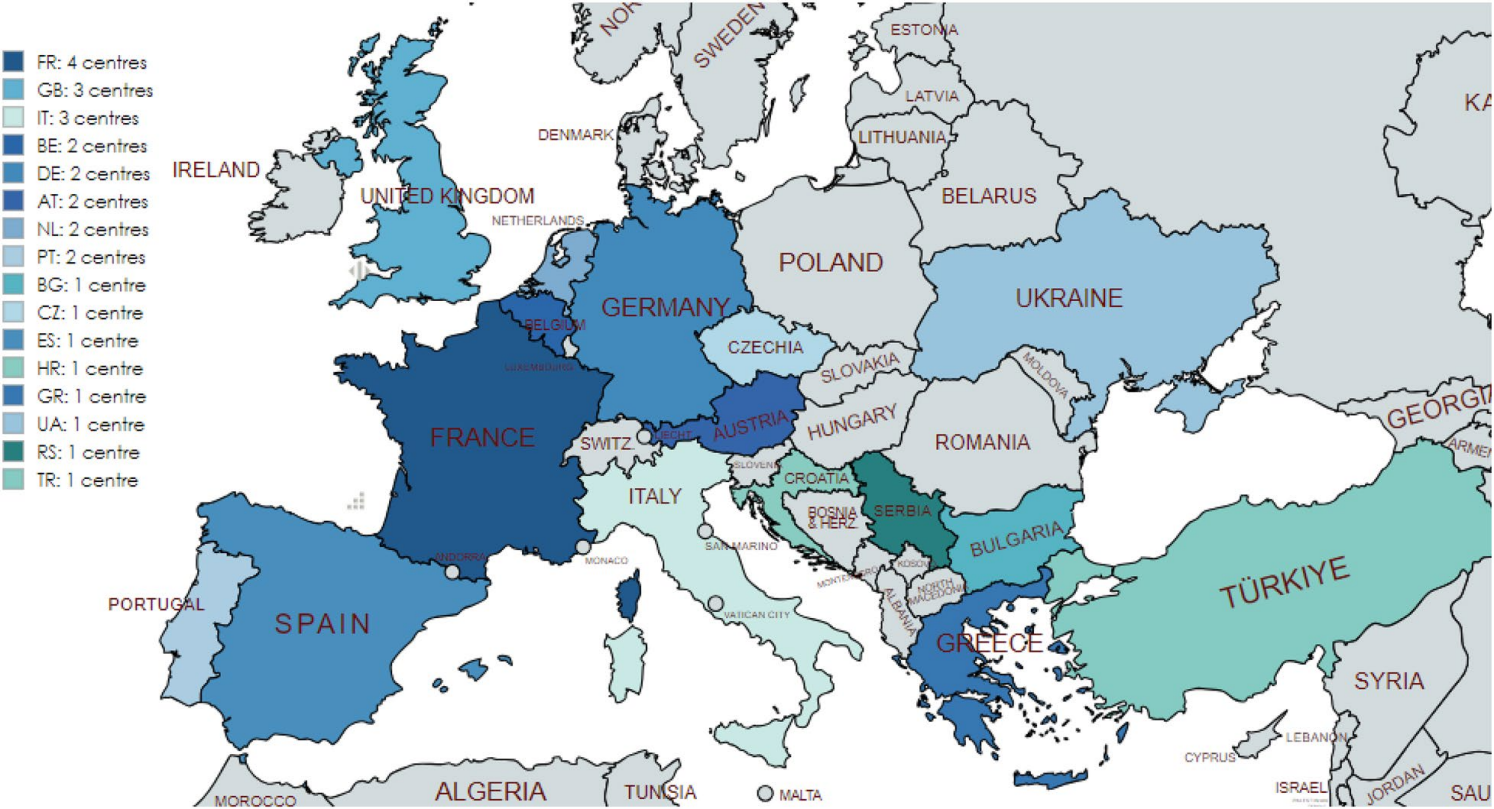
Core Registry centres including ERN BOND and non-ERN BOND members

AT, Austria; BE, Belgium; BG, Bulgaria; CZ, Czech Republic; ES, Spain; HR, Croatia; DE, Germany; FR, France; GB, the United Kingdom; GR, Greece; IT, Italy; NL, Netherlands; PT, Portugal; TR, Turkey; UA, Ukraine. Image created using MapChart.net.

Until July 2024, a total of 29 centres from 17 countries (four non-EU) had reported on the Core Registry: 1,245 cases (1,000 in bone dysplasia, 245—in mineral conditions). Of these 29 centres, three are in ERN BOND only, six are in Endo-ERN only, nine are in Endo-ERN and ERN BOND, and 11 are not related to ERNs (Fig. 5).

### Collaborative Governance for Improved Management

**Fig. 6:**
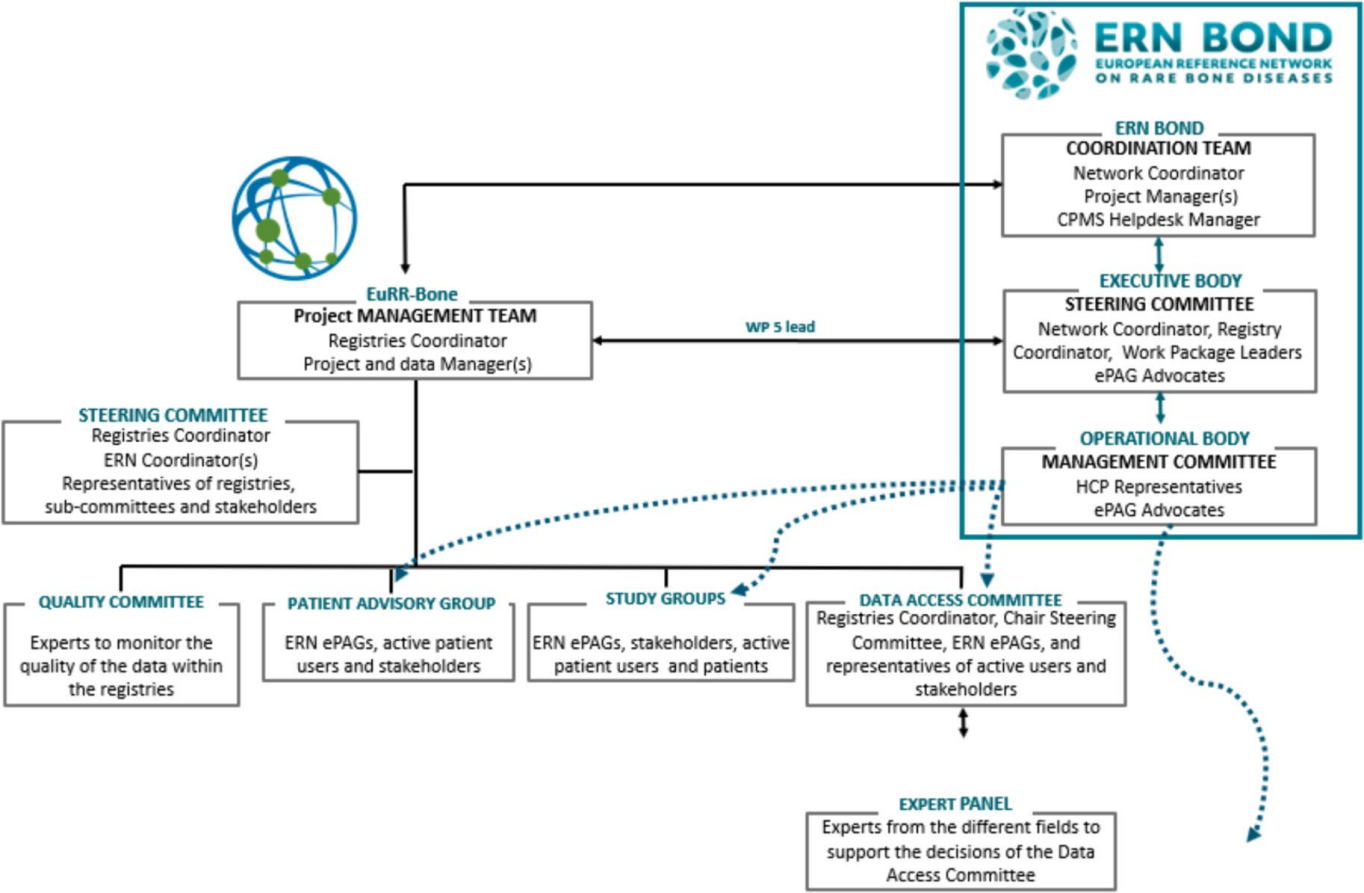
Overview of the collaborative governance of both projects

Since their inception, ERN BOND and EuRR-Bone have worked diligently to establish robust governing structures, prioritising transparency, patient inclusion, and interoperability. Although EuRR-Bone was separately funded from 2020 to 2023, this commitment is evident in the presence of coordinators from each entity within the governing bodies of the other, ensuring seamless interaction and mutual engagement (Fig. 6).

Additionally, all members of ERN BOND and ePAGs actively contribute to the initiatives of both organisations. From 2023 onwards, the registries were incorporated into the ERN structure as dedicated registries WPs. EuRR-Bone and ERN BOND collaboration has been the basis for the development of modules, demonstrating a shared commitment to enhancing patient care. The combined efforts of ERN BOND and EuRR-Bone registries, alongside dedicated governance policies, pose a significant advancement in managing RBMDs. This collaborative approach fosters a “win–win” ecosystem, promoting good practice standards within Europe. Through this partnership, several key benefits emerge such as the improved access to expert care and enhanced diagnosis. ERN BOND serves as a platform for healthcare professionals to share expertise, facilitating timely diagnosis for geographically dispersed patients facing distinct challenges or mobility difficulties. With the use of the e-REC platform, continuous monitoring can be facilitated and will allow the mapping of conditions and expert centres across Europe. This information can be useful for targeting centres for clinical patient management system discussions or clinical trial recruitment. Data from the Core Registry will inform the network on the natural history of RBMDs, identifying research gaps, help with the design of clinical trials, and support the development of novel therapies. By means of the registries, every HCP will be able to use validated patient-reported outcome measures and the bar can be set for care pathways.

By the active use of the registries and the participation in the different ERN BOND working groups, there will be enhanced access to information and participation in research initiatives. This will empower patients, positively impacting recruitment and retention within the registries and the network.

The network and registries’ joint dissemination activities amplify visibility across social media and websites, broadening the network and engaging with stakeholders. ERN BOND and EuRR-Bone promote joint training events, facilitate expertise exchange, educate young researchers and clinicians, and empower patients and families. In summary, the collaborative efforts of ERN BOND and EuRR-Bone represent a significant advancement in RBMDs management. Their partnership emphasises a holistic approach that prioritises patient needs, research advancement, and healthcare excellence across Europe.

### Future Directions

As the ERNs proceed into their next phase, collaborations will be built upon existing achievements, taking future directions for optimising RBMDs management through collaborative governance. ERN BOND will further engage its network with highly specialised healthcare professionals for further development of the registries and data entries. This will then allow the registries to provide ERN BOND the real-world data it requires to facilitate further improvement of care for patients with RBMDs. Furthermore, these data will facilitate more robust research in RBMDs and will foster wider collaboration.

**Fig. 7:**
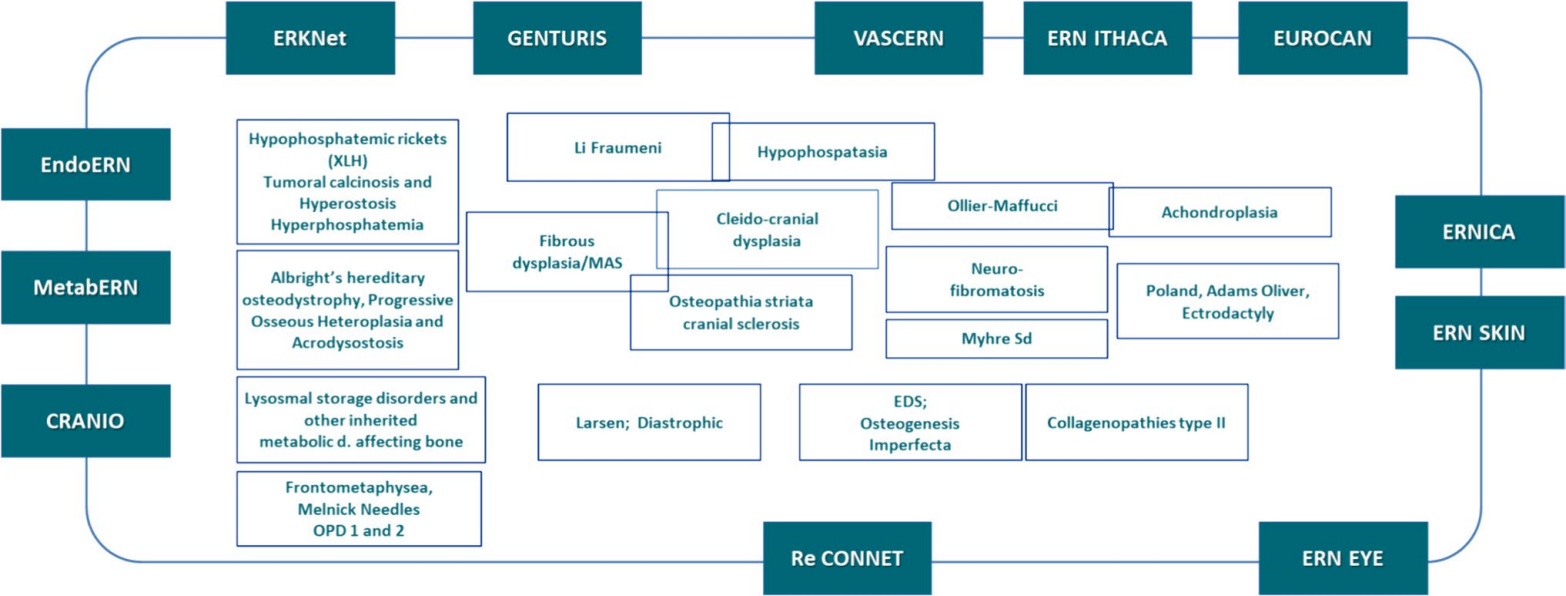
ERN BOND diseases overlapping with other ERNs

To reach these outcomes, the integration of the patient voice and journey has to stay secured allowing patient engagement and participation in research activities, including planning and decision-making when choosing and developing patient-reported outcome measures. As patients often have conditions that may be covered by several ERNs (Fig. 7), the interoperability of the registries as well as interaction between ERNs will be a matter of ongoing discussion.

Projects facilitating this interaction have been supported by the EU (Table 1). Examples of these initiatives are the European Rare Disease Research Coordination and Support Action consortium (ERICA) and the European Joint Programme on Rare Diseases (EJ PRD), both ending in 2024. New initiatives such as the European Rare Diseases Research Alliance (ERDERA) and the Joint Action on Integration of ERNs into National Healthcare Systems project (JARDIN) will ensure further collaborations within the coming years. However, securing ongoing funding for the ERNs and their registries is essential for their maintenance.

**Table 1:** Projects facilitating the interaction between ERNs. EJ PRD: European Joint Programme on Rare Diseases; ERICA: European Rare Disease Research Coordination and Support Action consortium; ERDERA: European Rare Diseases Research Alliance; JARDIN: Joint Action on Integration of ERNs into National Healthcare Systems; ERN: European Reference Network; WP: work package. *Projects without own website due to recent establishment

Initiative and weblink	Start and end date	Overall purpose	Interaction with registries	Interaction with ERNs
EJP RD	Jan 2019–Jul 2024	To create a comprehensive, sustainable ecosystem allowing a virtuous circle between research, care, and medical innovation	Offer training resources and support to ERN registries to integrate FAIR principles into their structure, e.g. FAIR data stewardship	Representation of ERNs in the governing board. EJPRD offers funded training workshops to ERN members. EJPRD mobility grant to allow exchange between ERN centres for research purposes
ERICA	May 2021–Jul 2024	To build on the strength of the individual ERNs and create a platform that integrates all ERNs research and innovation capacity	Coordination of activities to advance the development and integration of ERN-wide rare disease registries and their utilisation for joint research initiatives	Promotion of inter-ERN research activities. Participation of all ERNs in the WPs
ERDERA*	Sep 2024–Aug 2031	Promote European research in RD research and innovation, to support concrete health benefits to rare disease patients, through better prevention, diagnosis, and treatment	Use of population-based data derived from the registries for RD outcome research; integration of patient cohorts for natural history/standard of care reference studies; disease progression modelling and prognostic research	Through the ERN registries and the cross-border expertise on clinical rare disease entities
JARDIN*	Feb 2024–Jan 2027	To amplify the impact of ERNs, to improve access to timely diagnosis and appropriate treatment of RD patients and health professionals, and to support the long-term sustainability of the ERN system by contributing to the effective integration of ERN in the national health systems	Development of recommendations ensuring the interoperability of data structures on member state and ERN level	By supporting ERN-specific dissemination activities; promoting ERN-compliant governance models and practices for rare and complex diseases

Whilst significant progress has been made in recent years, certain areas still present challenges. Enhancing collaboration and mapping necessitates precise definitions and standardised ontologies. How diseases are coded in registries and hospitals can significantly impact the governance of rare diseases. Altering disease coding could disrupt retrospective data analyses and disease monitoring, with profound implications for rare conditions. Moreover, streamlining data collection through automation, whilst reducing the registration burden for healthcare professionals, is increasingly vital, particularly since countries are facing healthcare access constraints. Registries can potentially facilitate clinical benchmarking and mapping adherence to guidelines which can be used as quality indicators.

The combination of a clinical network for rare diseases with registries is unparalleled globally, significantly enhancing the provision of care and deepening understanding of rare conditions to unprecedented levels. This synergy enables further interactions with stakeholders, such as the European Medical Agency, pharmaceutical companies, patient associations, and governments. In the past years, the ERNs have been very successful in creating clinical networks dedicated to providing the best care to patients with rare conditions. In the coming years, ERN BOND will build upon these structures using, amongst other resources, the registries to increase our knowledge of and care for patients with RBMDs.
